# Oral Administration of the Endocannabinoid Anandamide during Lactation: Effects on Hypothalamic Cannabinoid Type 1 Receptor and Food Intake in Adult Mice

**DOI:** 10.1155/2017/2945010

**Published:** 2017-07-20

**Authors:** Carolina Aguirre, Valeska Castillo, Miguel Llanos

**Affiliations:** ^1^Unidad Docente Asociada, Ciencias de la Salud, Nutrición y Dietética, Pontificia Universidad Católica de Chile, Campus San Joaquín, Chile; ^2^Laboratorio de Nutrición y Regulación Metabólica, Instituto de Nutrición y Tecnología de los Alimentos (INTA), Universidad de Chile, Santiago, Chile

## Abstract

We have previously shown that administration of the endocannabinoid anandamide (AEA) during lactation leads to overweight, increased body fat accumulation, and insulin resistance in adult mice. This study was designed to elucidate if these effects are due to increased food intake, stimulated by an augmented abundance and binding ability of the hypothalamic cannabinoid type 1 receptor (CB1R). With this aim, male mice pups were treated with a daily oral dose of AEA during lactation. Adult mice were also treated with a single oral dose of AEA, to evaluate acute food intake during 4 h. At 21 and 160 days, CB1R protein abundance was calculated by western blot analysis. Capacity of hypothalamic membranes to specifically bind the radioligand ^3^[H]-CP55.940 was also measured. Western blots showed a 72% increase in CB1R abundance in AEA-treated 21-day-old mice, without differences in adult mice. Additionally, specific binding of ^3^[H]-CP55.940 to hypothalamic membranes from adult mice was significantly lower in those mice treated with AEA during lactation. Moreover, AEA did not stimulate acute food intake in both, AEA-treated and control mice. Results suggest that metabolic alterations found in adult mice because of AEA treatment during lactation are not associated with hypothalamic CB1R.

## 1. Introduction

The mechanism that regulates food intake is controlled by the hypothalamus, which is continuously informed about the nutritional and energy status of the body through peripheral and central orexigenic or anorexigenic signals. These signals include peripheral satiety messages and nerve inputs provided by adiposity, leptin, and intestinal peptides, while central messages are communicated by neuropeptides, monoamines, and endocannabinoids [1].

The endocannabinoid system is involved in food intake regulation through the expression and/or action of several hypothalamic anorexigenic and orexigenic mediators. Endocannabinoids activate cannabinoid type 1 and type 2 receptors (CB1R/CB2R). Type 1 receptors are colocalized with corticotrophin-releasing hormone (CRH) in the paraventricular nucleus (PVN), with melanin-concentrating hormone in the lateral hypothalamus, and with the preproorexin in the ventromedial hypothalamus [2, 3].

The CB1R gene deletion upregulates CRH, pointing to a tonic inhibition of the expression of this anorectic mediator by endocannabinoids [2]. Moreover, in the PVN, postsynaptic endocannabinoids, through CB1R, induce a retrograde inhibition of the glutamatergic release from neurons, thus mediating the rapid inhibition of corticosterone-induced CRH release [4]. Retrograde signalling exerted by endocannabinoids produced in postsynaptic depolarized neurons also inhibits the presynaptic release of GABA in the lateral hypothalamus and arcuate nucleus [5], confirming the role of the endocannabinoid system in the central circuits that regulate appetite.

Additionally, it has been suggested that endocannabinoids affect the motivational process towards food consumption by stimulating neuronal networks in the nucleus accumbens [6]. All these antecedents indicate that the endocannabinoid system is one of the key components in the central regulation of energy homeostasis.

Several studies in animal models have shown that administration of endocannabinoids stimulates food ingestion [7, 8]. Specifically, the administration of anandamide both centrally and peripherally in rodents stimulates food consumption [9]. In addition, this endocannabinoid is also able to stimulate food intake in satiated fish in a dose-dependent manner, with this effect being observed at concentrations as low as 1 pg/g of body weight [10]. Stimulation of CB1R with synthetic, high affinity agonists stimulates food intake even in satiated animals [11]. Pharmacological blockade of the receptor by systemic administration of SR141716A (Rimonabant), the first selective antagonist-inverse agonist of CB1R, reduces the stimulatory effect of the agonist and decreases both palatable food consumption in animals fed ad libitum and the normal food consumption in fasting animals [12].

Other evidences obtained in mice with a deletion of the gene encoding the CB1R (CB1R^−/−^) show that these mice are hypophagic and have less visceral and subcutaneous adipose tissue and greater lean mass when compared to native mice [2]. Additionally, when CB1R^−/−^ mice are subjected to a high fat diet, they are resistant to obesity, while native mice get obese to the same diet [13]. In previous studies, we have shown that administration of the endocannabinoid anandamide (AEA) during lactation induces overweight, fat accumulation, and metabolic alterations such as insulin resistance in adult CD1 mice, in concomitance to a higher expression of CB1R in visceral adipose tissue. However, it is not clear whether weight gain is due to a priming, long-term effect of AEA on its receptor in central nervous system, or in peripheral tissues such as the adipose tissue or both [14, 15].

With all these antecedents in mind, the aim of this study was to evaluate the effects of oral administration of AEA to male mice pups during lactation on the abundance and binding capacity of the hypothalamic CB1R, to relate it with acute AEA-stimulated and long-term unstimulated food intake.

## 2. Materials and Methods

All procedures performed in this study were approved by the Bioethics' Committee for Animal Experimentation of the Institute of Nutrition and Food Technology, University of Chile, Santiago, Chile.

### 2.1. Animals

Synchronously pregnant female CD1 mice were kept in the animal house under normal conditions of humidity and temperature (22–24°C), on a 12:12 h light-dark cycle. Animals had free access to purified tap water and food. A normal diet of 4 Kcal/g, equivalent to 2.8 assimilated Kcal/g (Champion Co., Santiago, Chile), was used during the whole study.

From day 16, pregnant female mice were daily examined at 9:00 and 19:00 h for the presence of pups. After 12–16 h since detection of pups, 6–8 litters of homogeneous size (12–14 pups) were put together and males separated from females. Afterwards, six male pups showing homogeneous weights were randomly selected and assigned to a substitute mother so that pups received random cross lactation. Animals were then assigned to one of the following groups:

1. Control mice: during the whole lactation (21 d), pups were removed daily from the home cage and weighed, and 1 *μ*l/g of body weight of soy oil was orally given.
2. AEA-treated mice: during the whole lactation (21 d), pups were removed daily from the home cage and weighed, and 20 *μ*g/g body weight of AEA (Sigma-Aldrich Co., St Luis, MO, USA) in soy oil (1 *μ*l/g body weight) was orally given.

At day 21 of age, animals were separated from their mothers, and groups of three animals were placed in new cages until 150 days of life; during this period, body weight and food intake were evaluated. Twenty-one-day-old and adult animals were then sacrificed according to the guidelines for rodent euthanasia provided by the American Medical Veterinary Association [16] and the hypothalamic area was extracted.

### 2.2. Food Intake

The amount of food eaten was recorded every 10 days. Amount of accumulated food intake per cage (three animals) from day 21 to day 150 was calculated by subtracting lost food inside the cage due to spilling out.

At 130 days of age, a group of control and AEA-treated mice were treated with a unique dose of 20 *μ*g/g body weight of AEA at the beginning of the dark cycle. Then, food intake was evaluated every one hour until 4 hours after treatment.

### 2.3. Western Blot of CB1R in Hypothalamus

For western blot procedures, hypothalamus from AEA-treated and control animals was homogenized (Heidolph homogenizer DIAX 600) in 500 *μ*l of RIPA buffer (25 mM Tris-HCl; pH 7.6; 150 mM NaCl; 1% sodium deoxycholate; 0.1% SDS) in the presence of protease inhibitor cocktail (Sigma-Aldrich, USA, P2714). The protein was separated in a 10% SDS-polyacrylamide gel (Mini protean III System; Bio-Rad, USA) and subsequently transferred overnight at 4°C to a PVDF membrane. Rabbit polyclonal antibody for CB1R was used as the primary antibody (Cayman chemical Co, USA) and as the secondary antibody, an enzyme-conjugated anti-rabbit antibody was used (Bio-Rad, USA). CB1R was visualized by chemiluminescence (Western lightning* Plus*-ECL, enhanced chemiluminescence substrate; Perkin Elmer). The obtained protein bands were normalized against *β*-actin expression and quantified using Gel-Pro Analyzer 3.1 programme, USA.

### 2.4. Hypothalamic Membrane Preparation

Hypothalamic membranes [17–19] were prepared as follows: hypothalamic tissue was homogenized with 50 mM Tris-HCl buffer pH 7.4 containing 1x concentration of a protease inhibitor cocktail (Sigma-Aldrich, USA, P2714). The homogenate was first centrifuged for 10 min at 1000 ×g at 4°C and the supernatant was then ultra-centrifuged for 30 min at 45.000 ×g, 4°C. The obtained pellet was resuspended in 1 ml of homogenization buffer and finally centrifuged for 5 min at 1.000 ×g at 4°C. The supernatant was quickly frozen in liquid nitrogen and stored at −80°C for no longer than a week, until use. Protein concentration was determined according to the DC Protein Assay Kit (Bio-Rad, USA, 500-0113-15) using bovine serum albumin (BSA) as a standard.

### 2.5. Binding of CP-55.940 to Membrane Preparations

Binding assay was performed with 40–50 *μ*g of membrane protein in 0.2 ml of a buffer pH 7.4 (50 mM Tris-HCl, 2 mM EDTA, 3 mM MgCl_2_, 5 mg/ml fatty acid free BSA) at 30°C. Binding was initiated by addition of 0.15 *μ*Ci ^3^[H]-CP 55.940, a ligand for CB1 and CB2 receptors (Perkin Elmer, Specific Activity 174.6 Ci/mmol; 4.3 nM final concentration). Reaction was stopped after 1 h with 4 ml of ice-cold washing buffer pH 7.4 (50 mM Tris-HCl, 1 mg/ml fatty acid free BSA) and immediately filtrated—under soft vacuum—through a GF/B glass-fibre filters (Whatman, England) previously soaked for 1 h in washing buffer. Filters were washed twice with 4 ml of ice-cold washing buffer and dried for 1 h at 37°C. Filters were finally placed in glass scintillation vials with 10 ml of Bray's liquid scintillation cocktail. Vials were maintained overnight at 4°C, then shaken and radioactivity evaluated by liquid scintillation spectrometry. Binding was expressed as fmol of ^3^[H]-CP-55.940/mg of protein. Nonspecific binding was determined in the presence of 4 *μ*M cold CP-55.940. Additional competence experiments were performed with 4 *μ*M anandamide to evaluate binding to CB1R. Experiments were carried out in triplicate and results represent data of 3–6 hypothalamuses from independent animals.

### 2.6. Statistical Analysis

Data were expressed as mean ± SEM. Shapiro-Wilk's and Levene tests were previously done to evaluate normal distribution of data and homogeneity of variances. To test difference between treatments Mann–Whitney *U* Test was performed. Statistical significance was set at *P* ≤ 0.05.

## 3. Results

### 3.1. Food Intake

**Figure 1:**
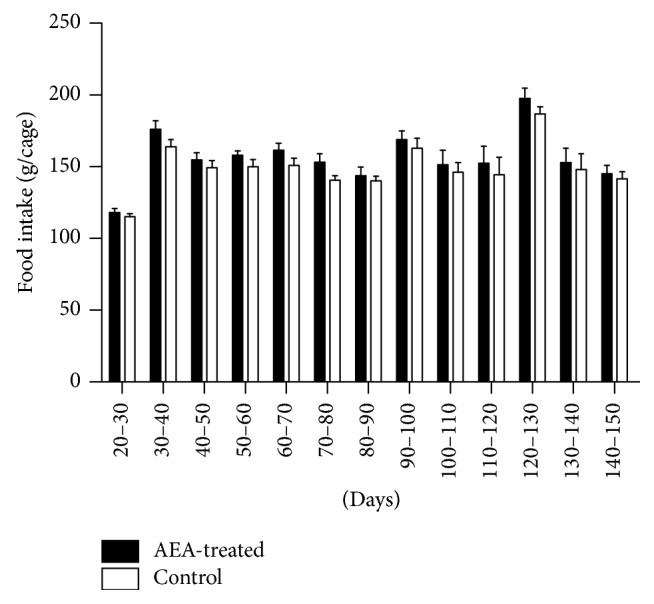
Food intake of control and AEA-treated animals during 10-day intervals (21–150 days old; mean ± SEM; *n* = 8 cages/group).

Figure 1 shows food intake of both groups of animals during 10 days intervals (21–150 days), results indicate that no difference in food intake was observed. However, when accumulated food consumption during 150 days was analyzed, AEA-treated mice ate 4.8% more food than control animals (2033 ± 32 g versus 1939 ± 28 g; mean ± SEM; *P* < 0.05; *n* = 8 cages per group containing 3 mice per cage).

At 130 days old, control and AEA-treated mice were again treated with 20 *μ*g/g body weight of AEA to evaluate the effect of this endocannabinoid over acute food intake during the first 4 hours after dose. No significant differences between both groups of mice were found in food intake at any 1 h time interval, nor in accumulated food intake during the whole period (Table 1).

### 3.2. Western Blot Analysis of CB1R in Hypothalamus

**Figure 2:**
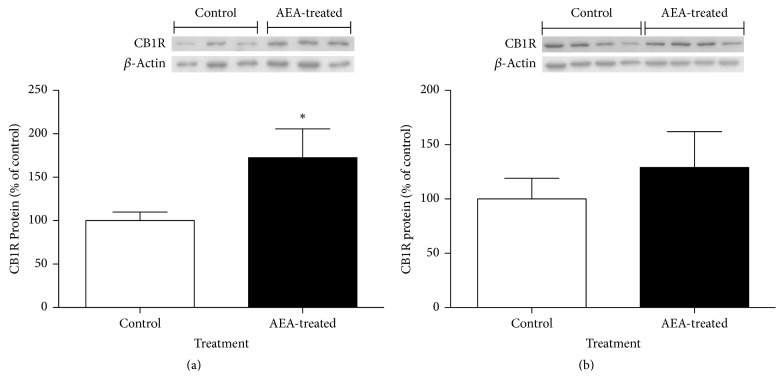
Relative levels of CB1R protein in hypothalamus of (a) 21-day-old and (b) adult AEA-treated and control mice. For densitometry quantification purposes, B-actin was used as loading control. Results are expressed as a percentage relative to the expression in control mice. (^*∗*^*P* < 0.05/^*∗∗*^*P* < 0.01 Mann–Whitney *U* test; mean ± SEM; *n* = 6/group).

Figure 2 shows CB1R protein expression in hypothalamus of 21- and 150-day-old control and AEA-treated mice. It is observed that 21-day-old AEA-treated mice had a significant 72% increase in CB1R abundance compared to control mice (*P* < 0.05; Figure 2(a)). Although 150-day-old AEA-treated mice had 29% higher expression, this value was not significantly different to control group (Figure 2(b)).

### 3.3. Binding of CP-55.940 to Hypothalamic Membrane Preparation

**Figure 3:**
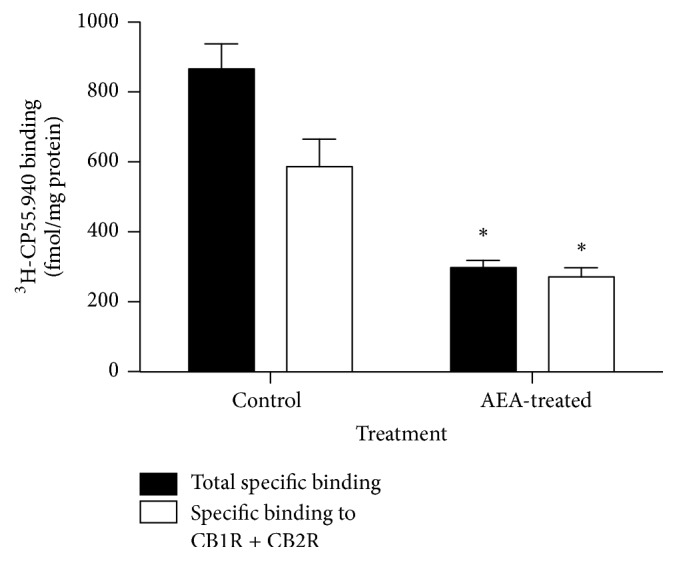
Specific binding of ^3^[H]-CP55.940 in hypothalamic membranes of control and AEA-treated 150-day-old mice (^*∗*^*P* < 0.05 Mann–Whitney *U* test; mean ± SEM; *n* = 3/group).

When assessing endocannabinoid receptors in hypothalamic membrane preparations, to evaluate their binding capacity to its radioactive ligand ^3^[H]-CP55.940, it is observed that 150-day-old AEA-treated mice have a significantly lower total specific binding than control mice (Figure 3). This difference is not observed in hypothalamic membrane preparations from 21-day-old mice (data not shown). It should pointed out that although the radioligand ^3^[H]-CP55.940 is able to bind with similar affinity to both CB1 and CB2 receptors, AEA shows a slightly more affinity for CB1R. However, 4 *μ*M AEA concentration used to displace ^3^[H]-CP55.940 might displace binding from both receptors, due to close Ki of AEA for CB1 (88 nM) and CB2 (268 nM). Consequently, displacement with AEA should indicate specific binding to both receptors.

## 4. Discussion

The present results indicate that mice treated during lactation with a daily oral dose of the endocannabinoid anandamide (AEA) show a significant increase in the hypothalamic CB1R protein abundance at 21 days of age, when compared to controls. Nevertheless, this difference in CB1R protein abundance is not maintained until adulthood because 150-day-old animals from both groups show similar hypothalamic CB1R levels. The greater body weight previously observed in AEA-treated mice [14] may be associated with a higher food intake, given the importance of the central endocannabinoid system in the mechanisms modulating food intake, where hypothalamic CB1R plays a key role [20]. However, the higher accumulated food intake over 150 days, found in AEA-treated animals, might not be associated with the levels of CB1R, which are similar to the CB1R level found in control mice. Additionally, 130-day-old AEA-treated animals and controls consumed the same amount of food in response to an acute, single oral dose of AEA, a fact that is concordant with the finding of the same CB1R levels in both groups. Interestingly, presence of cannabinoids receptors in hypothalamic membranes evidenced by its binding ability to CP-55940, a synthetic CB1R/CB2R agonist, is decreased in 150-day-old AEA-treated mice. Since total CB1R protein abundance is similar in both groups according to western blot results obtained, a differential, long-term programming of receptor compartmentalization due to AEA treatment during lactation may be suggested.

The higher cumulative food intake observed in AEA-treated mice, might be explained by another mechanism not directly involving abundance of CB1R in hypothalamic membranes. Thus, the explanation should be focused on the fact that those receptors should be activated by appropriate levels of endocannabinoids. In this regard, it is known that leptin exerts an inhibitory effect on hypothalamic endocannabinoid [21] production, leading to a decreased retrograde activation of CB1R, which finally results in a lower food intake. In this sense, it is known that obese mice with defective leptin signalling (*ob/ob* and* db/db*) show elevated levels of hypothalamic endocannabinoids [22], which are able to overactivate CB1R, regardless of its population. Furthermore, leptin administration decreases both food intake and endocannabinoid levels in wild type and* ob/ob* mice [22]. Since we have previously shown that AEA-treated animals have increased body weight mainly due to increased body fat content in addition to higher circulating levels of leptin [14, 15], it may be suggested that these mice could develop some level of chronic leptin resistance involved in the mild increased cumulative food intake observed. In this condition, hypothalamic endocannabinoid levels should be enough to chronically activate the decreased population of cannabinoids receptors found in hypothalamic membranes of AEA-treated mice, leading to the higher cumulative food intake observed in this study.

## Figures and Tables

**Table 1:** Food intake during the first 4 hours after a dose of AEA in adult mice.

Time interval	Food intake (g/mice)	
	Control	AEA-treated
0-1 h	1.03 ± 0.34	1.09 ± 0.32
1-2 h	1.56 ± 0.21	1.27 ± 0.31
2-3 h	1.24 ± 0.22	1.49 ± 0.11
3-4 h	1.19 ± 0.24	1.08 ± 0.13
0–4 h	5.02 ± 0.21	4.92 ± 0.49

Data represent mean ± SEM (*n* = 6).
